# Cancer mortality trends in an industrial district of Shanghai, China, from 1974 to 2014, and projections to 2029

**DOI:** 10.18632/oncotarget.21419

**Published:** 2017-09-30

**Authors:** Mi Li, Shuo Wang, Xue Han, Wenbin Liu, Jiahui Song, Hongwei Zhang, Jia Zhao, Fan Yang, Xiaojie Tan, Xi Chen, Yan Liu, Hui Li, Yibo Ding, Xiaoyu Du, Jianhua Yin, Rong Zhang, Guangwen Cao

**Affiliations:** ^1^ Department of Epidemiology, Second Military Medical University, Shanghai 200433, China; ^2^ Department of Chronic Diseases, Center for Disease Control and Prevention of Yangpu District, Shanghai 200090, China

**Keywords:** age-standardized mortality, cancer-caused life loss, pollution, lifestyle

## Abstract

We aimed to characterize the trends and projections of cancer mortalities in Yangpu, an industry restructuring district of Shanghai, China. With high-quality data from the death registration system, the authors analyzed the trends in cancer mortalities during 1974-2014 and their relationship with pollution control and socioeconomic improvements. Cancer burden was projected into 2029. During 1974-2014, cancer death accounted for 28.80% of all-cause death. The 5 leading causes of cancer death were cancers of the lung & bronchus, stomach, liver, colon & rectum, and esophagus. Age-standardized mortality of all cancers was higher in men than in women (153.1/10^5^ *vs*. 88.8/10^5^, p<0.001) and increased from 1974 to 1991 and decreased thereafter. The mortalities of cancers of the larynx, bladder, liver, nasopharynx, lung & bronchus, esophagus, lip oral & pharynx, stomach, kidney, and lymphoma were significantly higher in men than in women. Age-standardized mortalities of cancers of the esophagus, stomach, leukemia, female nasopharynx, female bladder, liver, and bone decreased especially after the 1990s, those of the colon & rectum, kidney, prostate, pancreas, breast, gallbladder, and ovary increased significantly. Lung cancer, breast cancer, colorectal cancer, and pancreas cancer in women and lung cancer, colorectal cancer, prostate cancer, and stomach cancer in men will be the leading causes of cancer death in 2025-2029. Cancer-caused life loss kept increasing since 2000. Conclusively, cancers associated with pollutions and infection decreased, especially after the 1990s, while those related to metabolic syndrome increased. These trends are related to closedown of polluted industries in the 1980s and lifestyle changes.

## INTRODUCTION

Cancer poses a major public health threat worldwide, with 14.9 million incident cancer cases and 8.2 million deaths in 2013 [1]. In low-income and middle-income countries, cancer becomes a major cause of death and will continue to increase as a percentage of deaths [2]. In mainland China, absolute numbers of incident cancer cases and cancer death keep increasing, from 3,586,200 incident cancer cases and 2,186,600 cancer deaths in 2012 to 4,292,000 incident cancer cases and 2,814,000 cancer deaths in 2015 [3, 4]. China is a big country with more than 1.3 billion people. Imbalance of economic development and diversity of lifestyles contribute to apparent differences in the incidences and mortalities of cancer types in different areas of China. Although estimates of cancer burden in China have been reported, the assumptions are limited to a snapshot of the patterns by cancer site in a given year [3–6] or to longitudinal situations of specific cancers and all cancer types for relatively short durations [7–11], making comparisons of long-term trends across all cancer types in accordance with economic development and lifestyle changes difficult.

Shanghai is a large modern city composed of commercial, agricultural, and industrial districts. Yangpu is an industrial district located in the northeastern corner (31°14’N, 121°29’E) of urban Shanghai and adjacent to lower reaches of Huangpu River, with a total of 1,243,757 residents permanently living in the 60.61 km^2^ of land in 2000. We selected Yangpu as the study field for three reasons. First, Yangpu remains the top among the national demonstration districts for the control of chronic diseases, therefore, historical record of death registration is intact and accurate. Second, population in Yangpu has been quite stable in the past 50 years. Third, highly polluting industries including coal-fired power generation, textiles, steel metallurgy, electroplating, and coal gas production had been introduced after the Second World War when the old municipal government was moved back to Yangpu, and had been shut down in the 1980s due to heavy industrial pollution, making Yangpu an ideal field for investigating the effect of the transition from industrial civilization to ecological civilization on cancer mortality. In the present study, we aimed to characterize the trends in cancer mortality from 1974 to 2014 in Yangpu and to give the projections to the year 2029. This study provides important references for controlling cancers in industry restructuring, middle-income societies.

## RESULTS

### Descriptive statistics

This study enrolled all registered permanent residents in Yangpu district during 1974-2014, with a total of 40,793,200 person-years. A total of 79,320 cancer death occurred, accounting for 28.80% of all death (275,427 total death). The residents aged 60 years or older during 1974-1984, 1985-1994, 1995-2004, and 2005-2014 accounted for 12.86%, 15.18%, 18.37%, and 22.87%, while cancer death in the 4 decades accounted for 26.12%, 26.65%, 28.51%, and 30.72% of all-cause death, respectively.

### Leading causes of cancer death among men and women

**Figure 1:**
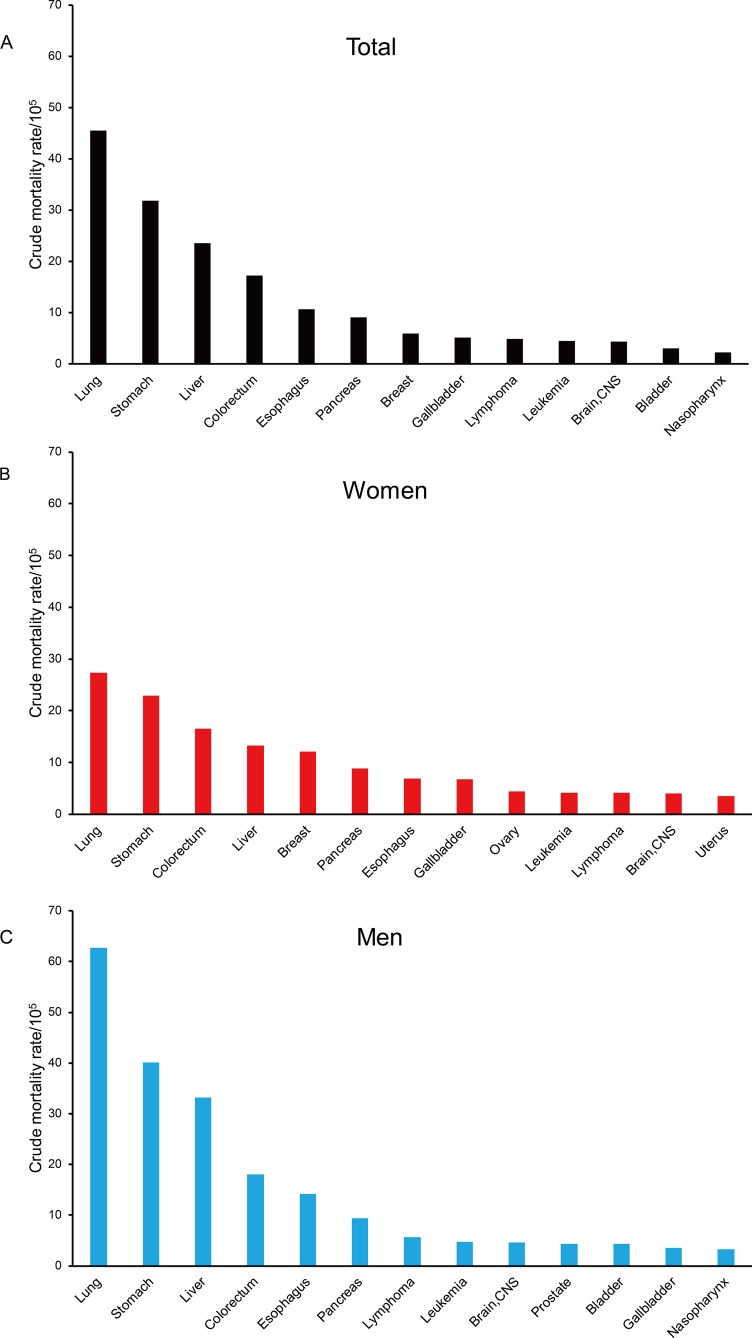
The major causes of cancer death in Yangpu, Shanghai, China during 1974-2014 **(A)** Total population; **(B)** female population; **(C)** male population.

The 5 leading causes of cancer death were cancers of the lung & bronchus, stomach, liver, colon & rectum, and esophagus, accounting for 67.50% of all cancer death. Among men, the 5 leading causes of cancer death accounted for 73.43% of all cancer death. Cancer of the breast, rather than esophagus, was one of the 5 leading causes of cancer death among women. Figure 1 shows the first 13 causes of cancer death in the total or sex-stratified populations. Age-standardized mortality rate for all cancers combined was significantly higher in men than in women (153.1/10^5^ *vs*. 88.8/10^5^, *P*<0.001). Crude mortalities of cancers of the larynx, bladder, liver, nasopharynx, lung & bronchus, esophagus, lip oral & pharynx, stomach, kidney, and lymphoma were significantly higher in men than in women, with a male-to-female ratio of 6.45, 2.63, 2.50, 2.46, 2.29, 2.05, 1.76, 1.75, 1.54, and 1.35, respectively; the mortalities of cancers of the breast, thyroid, and gallbladder were significantly higher in women than in men, with a male-to-female ratio of 0.01, 0.44, and 0.53, respectively (Supplementary Table 1).

### Average death age and age-specific mortality of selected cancers by sex

**Figure 2:**
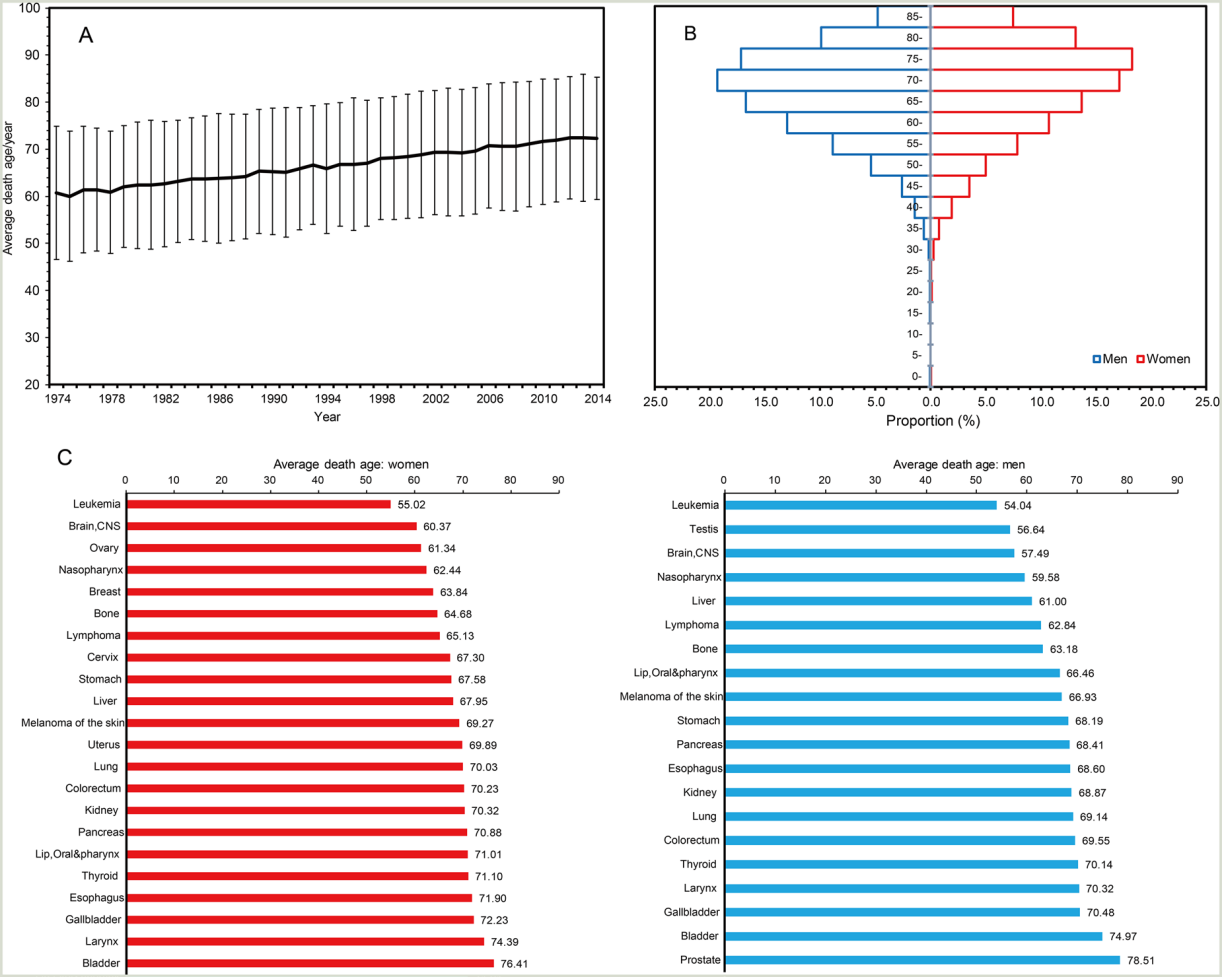
The average death age of permanent residents died of cancers in Yangpu, Shanghai, China, during 1974-2014

Average age of the residents died of cancers increased consecutively from 1974 to 2014 (Figure 2A). Cancer death peaked at 70-80 year old groups (70-75 years for men and 75-80 years for women) (Figure 2B). For most cancer types occurred in both sexes, men showed a significantly shorter life expectancy compared with women, except for those with stomach cancer (men *vs*. women: 68.2 *vs*. 67.6, *P*<0.001) (Figure 2C). Liver cancer was the leading cause of cancer death in men younger than 60 years old, followed by lung cancer and stomach cancer. The three cancer types were also the dominant causes of cancer death in men aged from 60 to 74 years. Stomach cancer was the leading cause of cancer death in women younger than 60 years, followed by lung cancer and breast cancer (Table 1). Most common cancer deaths occurred in the population aged from 60 to 74 years.

**Table 1:** Cancer deaths for selected cancers by age groups during 1974-2014

Cancer Site	Age, y					All
	<30	30-44	45-59	60-74	≥74	
Women						
Lung	11	161	886	2246	2101	5405
Stomach	64	304	843	1726	1592	4529
Colorectum	23	134	540	1181	1399	3277
Liver	13	115	540	1112	843	2623
Breast	10	212	824	701	636	2383
Pancreas	10	34	263	703	732	1742
Esophagus	3	11	174	626	551	1365
Gallbladder	3	17	179	511	626	1336
Ovary	19	69	347	271	167	873
Leukemia	139	117	175	222	181	834
Lymphoma	43	53	162	296	268	822
Brain, CNS	76	76	171	294	191	808
All sites	525	1528	5947	11714	11496	31210
Men						
Lung	12	290	2216	6457	4197	13172
Stomach	35	296	1588	3904	2618	8441
Liver	68	680	2529	2590	1097	6964
Colorectum	32	151	637	1477	1479	3776
Esophagus	1	56	601	1391	925	2974
Pancreas	4	54	397	893	625	1973
Lymphoma	66	83	256	479	296	1180
Leukemia	192	117	188	286	202	985
Brain, CNS	114	86	256	334	173	963
Prostate	1	2	26	243	646	918
Bladder	3	6	72	300	519	900
Gallbladder	2	22	116	304	309	753
All sites	671	2126	9966	20620	14727	48110

### Trends in cancer mortality during 1974-2014

**Figure 3:**
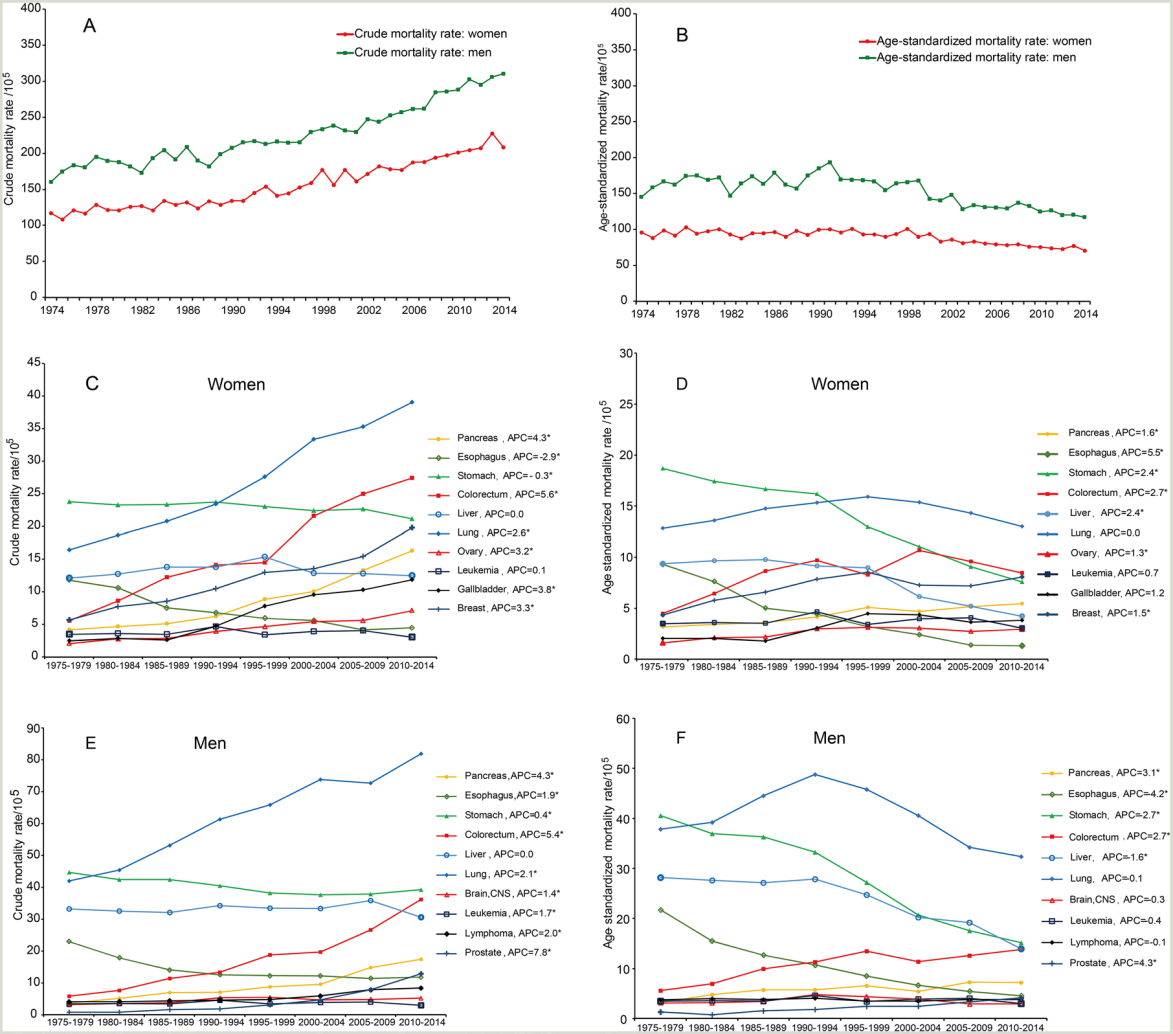
Trends in crude and age-standardized mortality rates for major cancer types in Yangpu, Shanghai, China from 1974 to 2014 **(A)** Trend in crude mortality rates of cancer in both sexes; **(B)** trend in age-standardized mortality rates of cancer in both sexes; **(C)** trend in crude mortality rates of major cancer type in women; **(D)** trend in age-standardized mortality rates of major cancer type in women; **(E)** trend in crude mortality rates of major cancer type in men; **(F)** trend in age-standardized mortality rates of major cancer type in men. APC: annual percentage change.

The rank order of leading causes of cancer death changed rather little over the 4 decades. The 5 leading causes were cancers of the stomach (crude mortality: 371.46/10^5^), lung & bronchus (331.84/10^5^), liver (249.97/10^5^), esophagus (176.60/10^5^), and colon & rectum (73.99/10^5^) during 1974-1984; cancers of the lung & bronchus (404.26/10^5^), stomach (328.42/10^5^), liver (239.18/10^5^), colon & rectum (127.49/10^5^), and esophagus (103.50/10^5^) during 1985-1994; cancers of the lung & bronchus (508.53/10^5^), stomach (305.62/10^5^), liver (240.74/10^5^), colon & rectum (186.46/10^5^), and pancreas (93.23/10^5^) during 1995-2004; cancers of the lung & bronchus (574.39/10^5^), stomach (303.04/10^5^), colon & rectum (287.75/10^5^), liver (229.90/10^5^), and pancreas (154.74/10^5^) during 2005-2014 (Supplementary Table 2). For all cancers combined, crude mortality rates increased steadily; however, the age-standardized mortality rates decreased significantly in both sexes during 1974-2014 (Figure 3A-3B). In women, colorectal cancer, pancreas cancer, ovary cancer, and breast cancer increased whereas esophageal cancer and stomach cancer decreased in the crude and age-standardized mortalities; lung cancer and gallbladder cancer increased in the crude mortalities, whereas liver cancer decreased in the age-standardized mortalities. In men, colorectal cancer, pancreas cancer, and prostate cancer increased whereas esophageal cancer and stomach cancer decreased in the crude and age-standardized mortalities; lung cancer, leukemia, lymphoma, and brain cancer increased in the crude mortalities, whereas liver cancer decreased in the age-standardized mortalities (Figure 3C-3F).

### Joinpoints of the age-standardized mortality rates during 1974-2014

Table 2 summarizes the results of joinpoint analyses for all cancers combined and the analyses of each cancer type. The age-standardized mortality rate for all cancers combined had increased until 1991 and thereafter have been decreasing significantly since 1991. Large increases in the age-standardized mortality rates of cancers of the lung & bronchus in both genders, bladder in men (Annual Percentage Change (APC)=2.3 during 1974-1993; APC=-4.5 after 1993), and brain & central nervous system (CNS) in men (APC=2.7 during 1974-1993; APC=-3.1 after 1994) to the 1990s have been reversing subsequently (Supplementary Table 3). Significant upward trends in the rates were observed for female breast cancer during 1974-1993 and for gallbladder cancer and ovary cancer during 1974-1998. Significant downward trends in the rates were evident for liver cancer during 1998-2014, bladder cancer during 1993-2014, and bone carcinoma during 1999-2014. The rates of esophageal cancer, stomach cancer, and leukemia persistently decreased, whereas those of cancers of the colon & rectum, pancreas, kidney, and prostate increased consistently during 1974-2014. Interestingly, the rates of cervical cancer increased during 1996-2014. No joinpoint was identified for the rates of nasopharyngeal carcinoma, larynocarcinoma, and lymphoma.

**Table 2:** Trends in age-standardized mortality rates in Yangpu, Shanghai, China, during 1974-2014

Cancer sites	APC (1974-2014)	Joinpoint Trend 1		Joinpoint Trend 2		Joinpoint Trend 3	
		Years	APC	Years	APC	Years	APC
All	-0.7[-0.9, -0.5]^a^	1974-1991	0.4[0.0,0.9]^a^	1991-2014	-1.5[-1.8, -1.3]^a^		
Lung	0.0[-0.4,0.3]	1974-1991	2.2[1.6,3.0]^a^	1991-2014	-1.7[-2.0, -1.3]^a^		
Stomach	-2.6[-3.0,-2.2]^a^	1974-1990	-1.0[-1.8,-0.1]^a^	1990-2014	-3.7[-4.1,-3.3]^a^		
Liver	-2.0[-2.6, -1.5]^a^	1974-1998	-0.5[-1.1,0.1]	1998-2014	-4.3[-5.3,-3.2]^a^		
leukemia	-0.8[-1.3,-0.2]^a^	1974-2014	-0.8[-1.3,-0.2]^a^				
Colorectum	2.7[1.9, 3.4]^a^	1974-1988	6.9[4.7,9.1]^a^	1988-2014	0.5[0.0,0.9]^a^		
Breast ^b^	1.5[0.6,2.4]^a^	1974-1993	3.6[2.0,5.2]^a^	1993-2014	-0.4[-1.3,0.5]		
Bladder	-1.0[-2.1,0.2]	1974-1993	1.9[-0.1,3.9]	1993-2014	-3.5[-4.7,-2.2]^a^		
Esophagus	-4.5[-4.8,-4.2]^a^	1974-2014^a^	-4.5[-4.8,-4.2]^a^				
Cervical^b^	1.3[-5.9,9.0]	1974-1993	2.1[-0.3,4.5]	1993-1996	-27.9[-73.4,95.5]	1996-2014	6.3[3.6,9.1]^a^
Brain, CNS	-0.2[-1.0,0.7]	1974-1995	2.5[1.3,3.7]^a^	1995-2014	-3.0[-4.3,-1.8]^a^		
Gallbladder	2.7[3.7,5.2]^a^	1974-1998	5.1[3.6,6.5]^a^	1998-2014	-0.8[-2.2,0.7]		
Pancreas	2.4[1.2,3.6]^a^	1974-1985	5.7[1.5,10.1]^a^	1985-2014	1.1[0.6,1.7]^a^		
Nasopharynx	-2.1[-5.4,1.2]	1974-2012	-0.3[-1.0,0.4]	2012-2014	-31.3[-65.1,35.1]		
Larynx	-0.5[-1.6,0.7]	1974-2014	-0.5[-1.6,0.7]				
Other thoracic organs	-0.7[-5.5,4.3]	1974-1989	-1.8[-8.0,4.9]	1989-1994	21.7[-13.6,71.4]	1994-2014	-4.8[-7.3,-2.3]^a^
Bone	-3.9[-5.4,-2.5]^a^	1974-1999	-0.4[-1.8,0.9]	1999-2014	-9.5[-12.6,-6.3]^a^		
Kidney	2.5[1.7,3.3]^a^	1974-2014	2.5[1.7,3.3]^a^				
Prostate^c^	4.3[3.5,5.0]^a^	1974-2014	4.3[3.5,5.0]^a^				
Lymphoma	-0.2[-0.7,0.3]	1974-2014	-0.2[-0.7,0.3]				
Ovary^b^	1.3[0.0, 2.6]^a^	1974-1998	2.9[1.2,4.6]^a^	1998-2014	-1.0[-3.1,1.2]		

^a^APC value is significantly different from Zero at alpha=0.05.

^b^APC value was calculated among women.

^c^APC value was calculated among men.

### Projections of the causes of cancer death up to 2029

**Figure 4:**
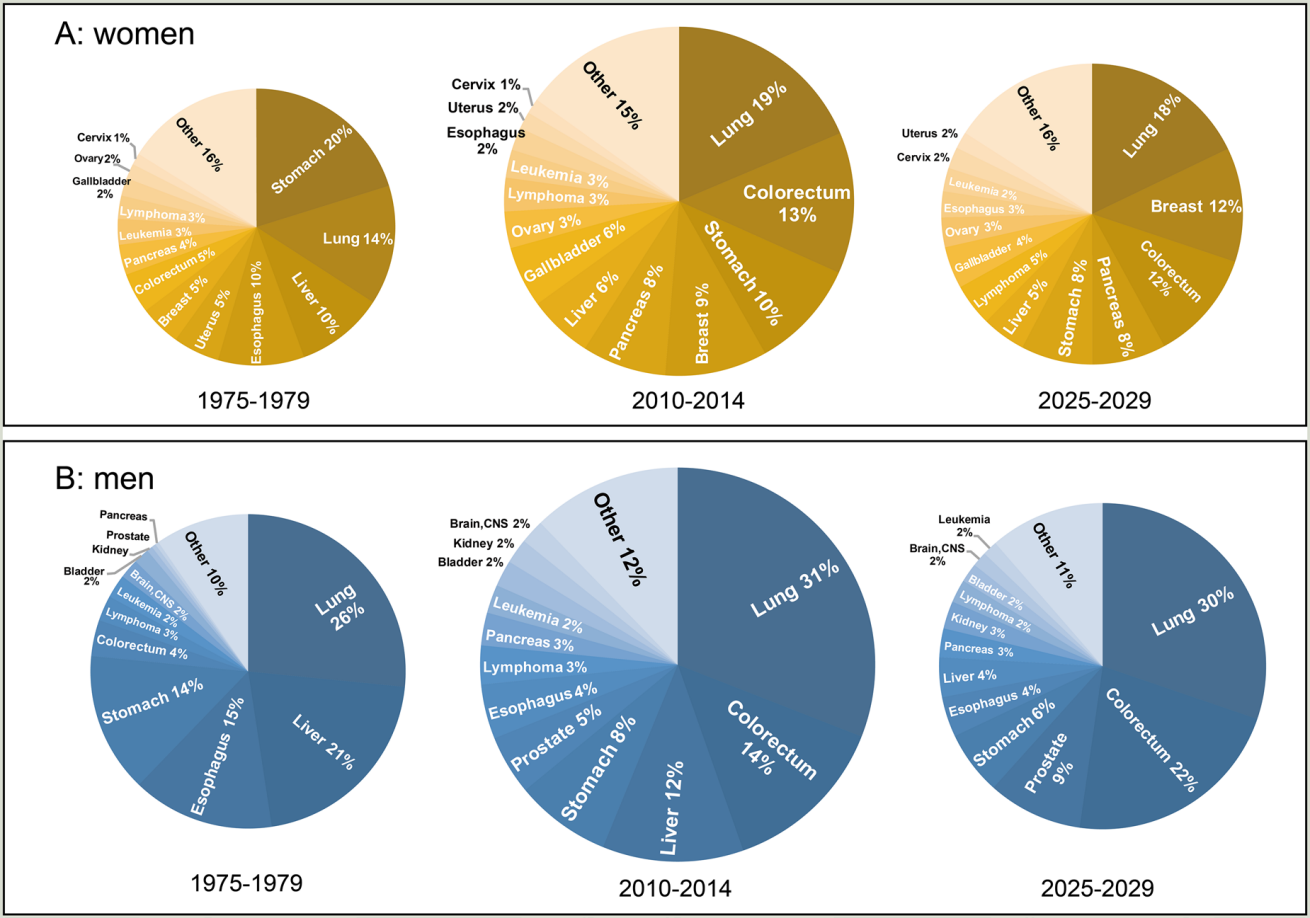
Pie charts of the most common causes of cancer death in women and men in 1975-1979, 2010-2014, and predicted in 2025-2029 The areas of the pies are proportional to the numbers of cases. **(A)** Scaled for women; **(B)** scaled for men.

The predicted number of cancer death in age groups from age-period-cohort model established using the data during 1975-2009 fitted well to the actual number of cancer death during 2010-2014 (*P*=0.971, Supplementary Table 4), with a determination coefficient of 99.33%, indicating that this model can be applied to project future cancer mortalities in Yangpu. A total of 15,549 women and 24,173 men are projected to die of cancer during 2015-2029. Lung cancer, colorectal cancer, breast cancer, pancreas cancer, and stomach cancer will be the major causes of cancer death, accounting for 18.50%, 12.17%, 10.87%, 8.58%, and 8.16% of total cancer death in women, respectively. Lung cancer, colorectal cancer, prostate cancer, stomach cancer, and liver cancer will be the major causes of cancer death, accounting for 31.04%, 19.22%, 8.03%, 6.61%, and 6.20% of total cancer death in men, respectively. The trends in the cause-specific mortalities are detailed in Supplementary Table 5. Figure 4 shows the proportionate distribution of different cancers among the totals in 1975-1979, 2010-2014, and 2025-2029. Actual number of cancer death in both sexes will decline in 2025-2029. For women, the rank order and percentage distribution change apparently over time, the biggest differences being the increases in beast and colorectal cancers and the decreases in stomach and liver cancers. Stomach cancer, the 1^st^ common cause (accounting for 20%) of cancer death in women before 1979, will be in the 5^th^ position (8% of cancer death in women) in 2025-2029, whereas breast cancer, which accounted for only 5% of cancer death in women before 1979, will comprise 12% of those in 2025-2029. For men, colorectal cancer and prostate cancer will increase apparently, whereas liver cancer, esophageal cancer, and stomach cancer will decrease remarkably. Colorectal cancer (the 5^th^ common cause of cancer death in men before 1979) is predicted to become the 2^nd^ common cause (accounting for 22%) of cancer death in men in 2025-2029. Liver cancer, the 2^nd^ common cause (accounting for 21%) of cancer death in men before 1979, will be the 6^th^ position (4% of cancer death in men) in 2025-2029. However, lung cancer remains to be the top cause of cancer death in both sexes.

**Figure 5:**
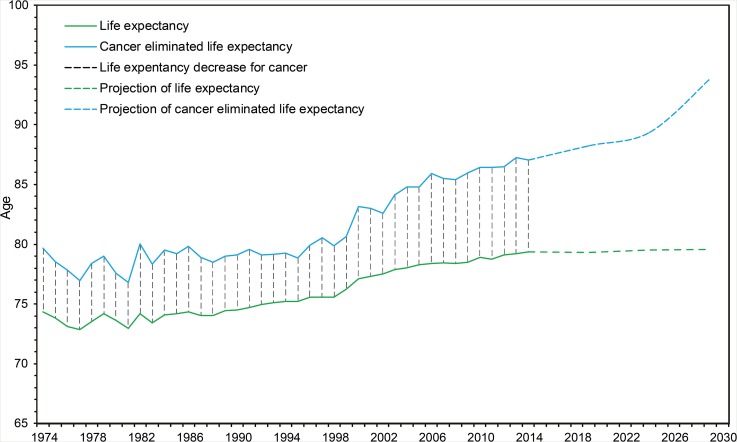
Trends and projections of annual life expectancy and cancer-eliminated life expectancy in Yangpu, Shanghai, China Trends: 1974-2014; projections: 2015-2029.

Trends (1974-2014) and projections (2015-2029) of annual life expectancy and cancer-eliminated life expectancy are shown in Figure 5. Cancer-caused life loss, calculated as cancer-eliminated life expectancy subtracted by life expectancy, was constant during 1974-1999 (*P*_trend_=0.143) and kept increasing from 2000 to 2014 (*P*_trend_<0.001). Cancer-caused life loss will keep increasing during 2015-2029.

## DISCUSSION

The updated estimates of cancer burden and long-term trends are critical to understanding the etiology, the effectiveness of prevention, early diagnosis, suitable treatment, and management of cancer in a given society. From the data of 22 population-based Chinese cancer registries during 2000-2011, the trends in age-standardized mortalities of major malignancies were quite consistent to the age-standardized incidences in both sexes [4], indicating that the effect of clinical activities on survival of cancer patients has not been improved. Thus, trends in the mortalities can reflect trends in the incidences of major malignancies in mainland China, a highly populated society with medium levels of medical services. In the present study, we selected Yangpu, an industry restructuring district of Shanghai, China, as the study field. The data from Yangpu can indicate the changes in cancer mortality during the transition from industrial civilization to ecological civilization in developing countries. We demonstrated that cancers of the lung & bronchus, stomach, liver, colon & rectum, and esophagus were the 5 leading causes of cancer death during 1974-2014. The 5 leading causes of cancer death were basically consistent to the national corresponding data in 2015, except that the rank order of colorectal cancer and esophageal cancer was counterchanged [4]. Cancers of the liver, lung, and stomach in men and cancers of the stomach, lung, and breast in women were the leading causes of cancer death for those aged before 60 years (Table 1), the young and working populations, indicating the control of these cancer types should have priority. Unlike most cancer types occurred in both sexes, women with stomach cancer survived shorter than did men (Figure 2), possibly because female stomach cancer is more associated with a younger age, poorly differentiated adenocarcinoma, and more signet ring cell carcinoma [12]. The mortalities for cancers of the larynx, bladder, liver, nasopharynx, lung, esophagus, lip oral & pharynx, stomach, kidney, and lymphoma were significantly higher in men than in women, and this result is quite consistent to sex disparity in the incidences of corresponding cancer types according to the national data in 2015 [4]. In addition to distinct opportunity of exposure to environmental risk factors such as tobacco smoking and working in polluting factories between men and women, some intrinsic factors such as sex hormone-related signaling pathways that facilitate cancer development in men may contribute to this sex disparity [12–15].

Cancer death accounted for 28.80% of total death in Yangpu during 1974-2014 and the proportion was quite correlated to that of residents aged 60 years or older in the 4 consecutive decades. Furthermore, crude mortality rates for all cancers combined increased and corresponding age-standardized mortalities decreased during 1974-2014 (Figure 3A-3B). These data indicate that aging contributes to increasing cancer deaths from 1974 to 2014. Improved social welfare and medical service benefited from economic development and the birth-control policy implemented in mainland China since the early 1970s did gradually alter age structure of this society, contributing to the aging-related profiling of cancer death.

A steady increase in the age-standardized mortality of all cancers combined up to around the year 1991 have been reversing subsequently (Table 2). This trend was predominantly contributed by the rates of cancers of the lung & bronchus in both sexes, bladder in men, and brain & CNS in men as well as other pollution-caused malignancies. Lung cancer was the 2^nd^ major cause of cancer death during 1974-1983. Thereafter, it became the leading cause of cancer death, possibly because highly polluting but economy-dependent industries introduced since 1945 were prosperous during the 1970s-1980s. These industries had been shut down in the 1980s due to heavy pollution, resulting in decreases in age-standardized mortalities of the pollution-related cancers after the 1990s. Although the age-standardized mortality decreased with a small slope, lung cancer remains the top cancer killer in Yangpu. This is possibly because the contribution of tobacco smoking to the mortality of lung cancer does not decrease apparently. In China, tobacco consumption has increased substantially since the 1980s, almost exclusively in men [16]. Over 50% of adult Chinese men were smokers in 2010 [17]. Tobacco smoking accounts for 23% of all cancer deaths in China [18]. Although stomach cancer was the top cause of cancer death during 1974-1983, persistent decreases in the crude and age-adjusted mortalities were evident (Supplementary Table 2 and Figure 3). Nevertheless, rapid decrease in the age-standardized mortality of stomach cancer occurred after 1990 (Table 2). This may be caused by wide use of refrigerator in households and enrichment of food supply since the early 1980s in Shanghai, as being demonstrated elsewhere [19]. Surprisingly, the crude and age-standardized mortalities of esophageal cancer rapidly declined (Supplementary Table 2 and Table 2). Consumption of hot food and beverages, tobacco smoking, and alcohol consumption are well-recognized risk factors, whereas diets rich in vegetables and fruits are protective factors of esophageal squamous cell carcinoma [20]. Chronic esophagitis such as reflux disease and Barrett's esophagus are major risk factors for the development of esophageal cancer [21]. We believe that sufficient supply of fresh and high quality food, diets rich in fresh vegetables and fruits, and public health education-improved healthy lifestyle like reducing consumption of hot food and beverages greatly reduce the prevalence of chronic esophagitis, thus contributing to rapid decline in the mortality of esophageal cancer. Age-adjusted mortality of liver cancer decreased significantly after 1998. Vaccination of hepatitis B virus initiated in 1987 in Shanghai contributes to this decrease in the rate of liver cancer, as evidenced in Taiwan [22]. Furthermore, factors contributing to the decrease in the age-standardized mortality of liver cancer include a reduction in the consumption of corn and peanut contaminated with aflatoxins in the hot and rainy geographic areas [23]. Increase in the age-adjusted mortalities of cancers of colon & rectum, pancreas, kidney, prostate, ovary, gallbladder, and breast should be related to the increasing prevalence of obesity and other metabolic syndrome including diabetes and hypertension [24–28], accompanying with economic growth, urbanization, and westernized lifestyle. Interestingly, the rate of cervical cancer started to increase after 1996, possibly due to the increase in sex-transmitted human papilloma virus infection after 1980 when the reform and opening to the outside world became a national policy in mainland China [29]. The decline in the age-standardized mortalities from all cancers combined and from major pollution-caused cancer types in Yangpu reflected the decrease in exposure to industrial pollution around 10 years ago. It seems too short to realize this reduction in cancer mortality, for the patients usually survive years since the occurrence. However, the major causes of cancer death in Yangpu are cancers of the lung & bronchus, stomach, liver, and esophagus, which are more aggressive than cancers of the colon & rectum, prostate, and breast, the major cancer types in developed world [1–6, 30]. Thus, reduction in industrial pollution and improvement in living standard at least 10 years may lead to the decrease in the mortalities of cancers caused by pollution, poverty, and chronic infection; economic growth, urbanization, and westernized lifestyle may increase the mortalities of cancers caused by metabolic syndrome.

Projections of cancer mortality provide an estimate of future burden of cancer and are fundamental to the process of planning for programs of cancer control as well as for setting priorities for future research. Age standardized mortality rates from cancer as a whole have been declining in Yangpu since 1991. Cancer of the stomach, esophagus, and liver consistently declined, whereas cancers of the colon & rectum, prostate, pancreas, and kidney increased in the past 41 years. The trends were projected to continue into the future. Cancers of the lung & bronchus, colon & rectum, prostate, and stomach in men and cancers of the lung & bronchus, breast, colon & rectum, and pancreas in women will be the top leading causes of cancer death in 2029, and the predicted top leading causes of cancer death in 2029 in Yangpu are similar to those in developed countries in 2008 [30, 31]. These evidence together with the truth that the 5 leading causes of cancer death in Yangpu during 1974-2014 were basically consistent to the corresponding national data in 2015, imply that the level of cancer control in China is at least 20 years behind developed counties. Cancer-caused life loss will keep increasing in the next 15 years. Therefore, public health efforts should be enforced to reduce tobacco consumption, decrease air pollution, provide healthy food and drinks, and encourage regular physical excise.

Our study has limitations. First, although death registration in Yangpu is the best in quality among China, it is difficult to characterize trends in the mortalities of rare cancer types such as laryngocarcinoma and cutaneum carcinoma due to a small population base. Second, the effect of industrial pollution on cancer mortality should be compromised by the improved medical service. Due to lack of the data concerning cancer incidences, it is difficult to figure out the benefit of the improved medical service.

Conclusively, the present study report the burden and trend of cancer death from 1974 to 2014 and the projections to 2029 in Yangpu, a district that can represent industry restructuring communities in developing world. Age-standardized mortalities of cancers of the lung & bronchus, bladder, brain & CNS, liver, stomach, and esophagus that are mainly caused by industrial pollution, poverty, and infection significantly decreased, especially after the 1990s; the mortalities of cancers of the colon & rectum, pancreas, prostate, and kidney that are associated with metabolic syndrome especially obesity and physical inactivity consistently increased from 1974 to 2014. This trend was projected to continue into the future. Cancers of the lung & bronchus, colon & rectum, prostate, and stomach in men and cancers of the lung & bronchus, breast, colon & rectum, and pancreas in women will be the leading causes of cancer death in 2029, and these malignancies requires public heath interventions in advance.

## MATERIALS AND METHODS

### Data collection

Information of cancer death during 1974-2014 was derived from the well-operated death registration system, covering all permanent residents in Yangpu, Shanghai. Cases were classified according to the 9^th^ (1974-2001) and 10^th^ (2002-2014) edition of International Classification of Disease codes. Yearly demographic data from 1994 to 2014 were obtained from the Public Security Bureau of Yangpu district. We did not extract personal information according to the regulations approved by the review board of Second Military Medical University.

### Statistical analysis

Temporal trends in the mortalities from 1974 to 2014 were examined by fitting joinpoint models (Version 4.3.1.0, Bethesda, MD) to the log-transformed, crude rates and age-adjusted rates standardized according to the world standard population [32, 33]. The trend was expressed as the APC and the Z test was employed to assess if the APC was statistically different from zero. The term “increase” or “decrease” was applied to describe the trend when APC of the trend was statistically significant (*P*<0.05). Cancer mortality was predicted by age-period-cohort model [34–36] and extrapolated out to 2029. Population number of each age group during 2015-2029 was predicted using Leslie matrix model [37]. To evaluate the effect of this model on predicting cancer mortality, we applied the mortalities during 1975-2009 to fit the model and then validated the model using the data from 2010 to 2014. A generalized linear model was applied to evaluate the consistency between actual value and predicted value. Life expectancy and cause-eliminated life expectancy referred to those of birth group and were calculated using the abridged life table [38]. Abridged life table was also used to calculate life expectancy and cause-eliminated life expectancy during 2015-2029. Numbers of all-cause death, cancer death and population data during 2015-2029, which were needed in the abridged life table, were obtained from the age-period-cohort model and the Leslie matrix model as previously described [37]. Life loss was calculated as subtracting life expectancy from cause-eliminated life expectancy. The trend of life loss was evaluated by piecewise linear fitting method [39]. We conducted the age-period-cohort model analysis using Norpred package and piecewise linear fitting analysis using segmented package in R software (Version 3.3.3). Basic statistical analysis was conducted using SAS9.4 software (SAS, NC). A *P* value of <0.05 in two sides was considered as statistically significant.

## SUPPLEMENTARY MATERIALS TABLES









## Abbreviations

APC: Annual Percentage Change
