# Supplementary material for: Cancer mortality trends in an industrial district of Shanghai, China, from 1974 to 2014, and projections to 2029
Source: Oncotarget. 2017 Sep 30;8(54):92470–82. doi: 10.18632/oncotarget.21419 (PMC5696197; doi:10.18632/oncotarget.21419)
Supplement: Supplementary file 1 [file oncotarget-08-92470-s001.pdf]

Cancer mortality trends in an industrial district of Shanghai, China, from 1974 to 2014, and projections to 2029

SUPPLEMENTARY MATERIALS

Supplementary Table 1: Difference in crude mortality rate of each cancer type between men and women during 1974-2014

Cancer sites	Crude mortality rate in men (/10^5)	Crude mortality rate in women (/10^5)	Ratio (Men/women)	Statistical tests[a]
Lip, Oral & pharynx	1.57	0.89	1.76	u=6.20, p<0.001
Nasopharynx	3.25	1.32	2.46	u=12.75, p<0.001
Esophagus	14.15	6.9	2.05	u=22.48, p<0.001
Stomach	40.16	22.9	1.75	u=31.11, p<0.001
Colorectum	17.97	16.57	1.08	u=3.40, p<0.001
Liver	33.14	13.26	2.50	u=41.58, p<0.001
Gallbladder	3.58	6.76	0.53	u=-41.12, p<0.001
Pancreas	9.39	8.81	1.07	u=1.94, p=0.052
Larynx	1.87	0.29	6.45	u=15.18, p<0.001
Lung	62.67	27.33	2.29	u=53.37, p<0.001
Other thoracic organs	0.98	0.66	1.48	u=3.64, p<0.001
Bone	2.19	1.89	1.16	u=2.10, p=0.036
Melanoma of the skin	0.32	0.37	0.86	u=-0.95, p=0.342
Kidney	2.46	1.6	1.54	u=6.05, p<0.001
Bladder	4.28	1.63	2.63	u=15.45, p<0.001
Brain, CNS	4.58	4.09	1.12	u=2.41, p=0.016
Thyroid	0.35	0.79	0.44	u=-5.87, p<0.001
Lymphoma	5.61	4.16	1.35	u=6.65, p<0.001
Leukemia	4.69	4.22	1.11	u=2.25, p=0.025
others	11.04	11.14	0.99	u=-0.31, p=0.760

[a]u value was calculated according to Poisson distribution.

Supplementary Table 2: Trends in the crude mortality rate of each cancer type during 1974-1984, 1985-1994, 1995-2004, and 2005-2014

See Supplementary File 1

Supplementary Table 3: Trends in age-standardized mortality rates stratified by sex during 1974-2014

See Supplementary File 2

Supplementary Table 4: The concordance of predicted number with actual number of cancer death during 2010-2014

Age group	Actual cases [a]	Predicted cases [a]
1-4	9	14.34
5-9	4	4.23
10-14	5	8.11
15-19	12	9.09
20-24	29	21.96
25-29	27	36.72
30-34	38	43.35
35-39	60	78.98
40-44	114	136.10
45-49	273	266.73
50-54	860	836.26
55-59	1493	1518.96
60-64	1473	1584.43
65-69	1110	1217.56
70-74	1295	1317.31
75-79	2378	2315.62
80-84	2604	2472.52
85-	2151	1797.25

[a]Difference was not significant (χ^2=0.01, *P*= 0.971).

Supplementary Table 5: Projected number of cancer death during 2015-2019, 2020-2024, and 2025-2029 by sex

See Supplementary File 3
