# Supplementary material for: Cancer mortality trends in an industrial district of Shanghai, China, from 1974 to 2014, and projections to 2029
Source: Oncotarget. 2017 Sep 30;8(54):92470–82. doi: 10.18632/oncotarget.21419 (PMC5696197; doi:10.18632/oncotarget.21419)
Supplement: Supplementary file 2 [file oncotarget-08-92470-s002.doc]

**Supplementary Table 2:** Trends in the crude mortality rate of each cancer type during 1974-1984, 1985-1994, 1995-2004, and 2005-2014

| **Cancer sites** | **1974-1984** | | |  | **1985-1994** | | |  | **1995-2004** | | |  | **2005-2014** | | |
| --- | --- | --- | --- | --- | --- | --- | --- | --- | --- | --- | --- | --- | --- | --- | --- |
|  | **Mortality rate (/10^5^)** | **Proportion (%)** | **Rank** |  | **Mortality rate (/10^5^)** | **Proportion (%)** | **Rank** |  | **Mortality rate (/10^5^)** | **Proportion (%)** | **Rank** |  | **Mortality rate (/10^5^)** | **Proportion (%)** | **Rank** |
| Lip, Oral & pharynx | 10.00 | 0.59 | 19 |  | 9.93 | 0.58 | 20 |  | 10.97 | 0.55 | 21 |  | 18.88 | 0.78 | 18 |
| Nasopharynx | 19.87 | 1.17 | 15 |  | 20.91 | 1.22 | 15 |  | 25.93 | 1.29 | 14 |  | 26.62 | 1.1 | 17 |
| Esophagus | 176.60 | 10.44 | 4 |  | 103.50 | 6.04 | 5 |  | 91.09 | 4.54 | 7 |  | 80.04 | 3.3 | 9 |
| Stomach | 371.46 | 21.96 | 1 |  | 328.42 | 19.18 | 2 |  | 305.62 | 15.22 | 2 |  | 303.04 | 12.5 | 2 |
| Colorectum | 73.99 | 4.37 | 6 |  | 127.49 | 7.45 | 4 |  | 186.46 | 9.28 | 4 |  | 287.75 | 11.87 | 3 |
| Liver | 249.97 | 14.78 | 3 |  | 239.18 | 13.97 | 3 |  | 240.74 | 11.99 | 3 |  | 229.90 | 9.48 | 4 |
| Gallbladder | 19.75 | 1.17 | 16 |  | 30.07 | 1.76 | 12 |  | 62.00 | 3.09 | 9 |  | 87.50 | 3.61 | 7 |
| Pancreas | 45.99 | 2.72 | 7 |  | 63.99 | 3.74 | 7 |  | 93.23 | 4.64 | 6 |  | 154.74 | 6.38 | 6 |
| Larynx | 7.62 | 0.45 | 21 |  | 9.35 | 0.55 | 21 |  | 12.36 | 0.62 | 20 |  | 14.55 | 0.6 | 20 |
| Lung | 331.84 | 19.61 | 2 |  | 404.26 | 23.61 | 1 |  | 508.53 | 25.32 | 1 |  | 574.39 | 23.69 | 1 |
| Other thoracic organs | 5.12 | 0.3 | 22 |  | 7.03 | 0.41 | 23 |  | 10.88 | 0.54 | 22 |  | 9.76 | 0.4 | 23 |
| Bone | 25.00 | 1.48 | 13 |  | 21.49 | 1.25 | 14 |  | 25.00 | 1.24 | 15 |  | 13.08 | 0.54 | 22 |
| Melanoma of the skin | 1.75 | 0.1 | 25 |  | 2.02 | 0.12 | 25 |  | 3.44 | 0.17 | 25 |  | 6.36 | 0.26 | 24 |
| Breast | 36.00 | 2.13 | 10 |  | 46.06 | 2.69 | 8 |  | 64.04 | 3.19 | 8 |  | 87.41 | 3.6 | 8 |
| Cervix | 11.87 | 0.7 | 18 |  | 13.40 | 0.78 | 18 |  | 6.97 | 0.35 | 23 |  | 13.36 | 0.55 | 21 |
| Uterus | 28.75 | 1.7 | 12 |  | 13.88 | 0.81 | 17 |  | 13.66 | 0.68 | 19 |  | 15.20 | 0.63 | 19 |
| Ovary | 13.25 | 0.78 | 17 |  | 16.38 | 0.96 | 16 |  | 24.17 | 1.2 | 16 |  | 31.13 | 1.28 | 16 |
| Prostate | 4.25 | 0.25 | 24 |  | 8.87 | 0.52 | 22 |  | 20.26 | 1.01 | 18 |  | 52.69 | 2.17 | 12 |
| Testis | 1.00 | 0.06 | 26 |  | 0.87 | 0.05 | 26 |  | 1.02 | 0.05 | 26 |  | 0.64 | 0.03 | 26 |
| Kidney | 8.50 | 0.5 | 20 |  | 11.76 | 0.69 | 19 |  | 21.19 | 1.06 | 17 |  | 38.23 | 1.58 | 15 |
| Bladder | 22.87 | 1.35 | 14 |  | 26.60 | 1.55 | 13 |  | 31.60 | 1.57 | 13 |  | 38.96 | 1.61 | 14 |
| Brain, CNS | 32.87 | 1.94 | 11 |  | 41.63 | 2.43 | 10 |  | 50.84 | 2.53 | 10 |  | 48.63 | 2.01 | 13 |
| Thyroid | 5.00 | 0.3 | 23 |  | 5.69 | 0.33 | 24 |  | 6.13 | 0.31 | 24 |  | 5.99 | 0.25 | 25 |
| Lymphoma | 39.62 | 2.34 | 9 |  | 39.32 | 2.3 | 11 |  | 48.80 | 2.43 | 11 |  | 69.27 | 2.86 | 10 |
| Leukemia | 41.25 | 2.44 | 8 |  | 42.02 | 2.45 | 9 |  | 41.64 | 2.07 | 12 |  | 55.63 | 2.29 | 11 |
| Other | 107.61 | 6.36 | 5 |  | 78.25 | 4.57 | 6 |  | 101.97 | 5.08 | 5 |  | 161.28 | 6.65 | 5 |
| Total | 1691.81 | 100 | / |  | 1712.35 | 100 | / |  | 2008.54 | 100 | / |  | 2425.05 | 100 | / |
