# Supplementary material for: Cancer mortality trends in an industrial district of Shanghai, China, from 1974 to 2014, and projections to 2029
Source: Oncotarget. 2017 Sep 30;8(54):92470–82. doi: 10.18632/oncotarget.21419 (PMC5696197; doi:10.18632/oncotarget.21419)
Supplement: Supplementary file 3 [file oncotarget-08-92470-s003.doc]

**Supplementary Table 3:** Trends in age-standardized mortality rates stratified by sex during 1974-2014

| **Cancer sites** | **Gender** | **APC (1974-2014)** | **Joinpoint Trend 1** | |  | **Joinpoint Trend 2** | |  | **Joinpoint Trend 3** | |
| --- | --- | --- | --- | --- | --- | --- | --- | --- | --- | --- |
|  |  |  | **Years** | **APC** |  | **Years** | **APC** |  | **Years** | **APC** |
| All cancer | Female | -0.7[-1.0, -0.5] ^a^ | 1974-1997 | -0.1[-0.3, 0.2] |  | 1997-2014 | -1.7[-2.1, -1.3] ^a^ |  |  |  |
|  | Male | 1.3[0.0,2.6] ^a^ | 1974-1998 | 2.9[1.2,4.6] ^a^ |  | 1998-2014 | -1.0[-3.1,1.2] |  |  |  |
| Lung | Female | -0.0[-0.5, 0.6] | 1974-1997 | 1.2[0.5, 2.0] ^a^ |  | 1997-2014 | -1.5[-2.4, -0.7] ^a^ |  |  |  |
|  | Male | -0.1[-0.6, 0.3] | 1974-1991 | 2.6[1.7, 3.5] ^a^ |  | 1991-2014 | -2.1[-2.5, -1.7] ^a^ |  |  |  |
| Stomach | Female | -2.4[-3.0,-1.9] ^a^ | 1974-1990 | -0.8[-1.9,0.3] |  | 1983-1986 | -3.5[-4.1,-2.9] ^a^ |  |  |  |
|  | Male | -2.7[-3.2,-2.2] ^a^ | 1974-1990 | -0.9[-1.9,0.1] |  | 1983-1986 | -3.9[-4.4, -3.4] ^a^ |  |  |  |
| Liver | Female | -2.4[-3.1,-1.8] ^a^ | 1974-1996 | -0.4[-1.3,0.5] |  | 1998-2014 | -4.9[-6.0,-3.8] ^a^ |  |  |  |
|  | Male | -1.6[-2.2,-1.0] ^a^ | 1974-1991 | 0.5[-0.7,1.7] |  | 1991-2014 | -3.1[-3.8,-2.4] ^a^ |  |  |  |
| leukemia | Female | -0.7[-2.9,1.6] | 1974-1993 | 1.3[-1.0, 3.6] |  | 1993-2009 | -0.9[-3.4,1.8] |  | 2009-2014 | -7.1[-19.6,7.5] |
|  | Male | -0.4[-1.1,0.4] | 1974-2014 | -0.4[-1.1,0.4] |  |  |  |  |  |  |
| Colorectum | Female | 2.7[0.3,5.1] ^a^ | 1974-1980 | 16.9[1.0,35.4] ^a^ |  | 1980-2004 | 1.5[0.4,2.7] ^a^ |  | 2004-2014 | -2.4[-5.2,0.4] |
|  | Male | 2.7[1.3,4.2] ^a^ | 1974-1998 | 4.5[3.4,5.6] ^a^ |  | 1988-2003 | -5.8[-14.8,4.1] |  | 2003-2014 | 3.0[1.0,4.9] ^a^ |
| Breast | Female | 1.5[0.6,2.4] ^a^ | 1974-1993 | 3.6[2.0,5.2] ^a^ |  | 1993-2014 | -0.4[-1.3,0.5] |  |  |  |
| Bladder | Female | -1.3[-2.2,-0.3] ^a^ | 1974-2014 | -1.3[-2.2,-0.3] ^a^ |  |  |  |  |  |  |
|  | Male | -1.1[-2.4,0.2] | 1974-1993 | 2.3[0.0,4.6] ^a^ |  | 1993-2014 | -4.5[-6.1,-3.0] ^a^ |  |  |  |
| Esophagus | Female | -5.5[-6.1,-5.0] ^a^ | 1974-2014 ^a^ | -5.5[-6.1,-5.0] ^a^ |  |  |  |  |  |  |
|  | Male | -4.2[-4.5,-3.8] ^a^ | 1974-2014 ^a^ | -4.2[-4.5,-3.8] ^a^ |  |  |  |  |  |  |
| Cervical | Female | 1.3[-5.9,9.0] | 1974-1993 | 2.1[-0.3,4.5] |  | 1993-1996 | -27.9[-73.4,95.5] |  | 1996-2014 | 6.3[3.6,9.1] ^a^ |
| Brain, CNS | Female | -0.2[-1.6,1,1] | 1974-1995 | 2.8[0.7,5.0] ^a^ |  | 1995-2014 | -3,5[-3.5,-1.7] ^a^ |  |  |  |
|  | Male | -0.3[-1.6,1.1] | 1974-1994 | 2.7[0.6,4.8] ^a^ |  | 1994-2014 | -3.1[-4.9.-1.3] ^a^ |  |  |  |
| Gallbladder | Female | 1.2[-1.0,3.5] | 1974-1988 | -0.6[-5.0,4.1] |  | 1988-1997 | 10.4[3.2,18.1] ^a^ |  | 1997-2014 | -1.9[-3.5,-0.2] ^a^ |
|  | Male | 4.0[2.0,6.1] ^a^ | 1974-1991 | 8.8[4.1,13.7] ^a^ |  | 1991-2014 | 0.7[-0.7,2.0] |  |  |  |
| Pancreas | Female | 1.6[1.1,2.0] ^a^ | 1974-2014 | 1.6[1.1,2.0] ^a^ |  |  |  |  |  |  |
|  | Male | 3.1[1.0,5.3] ^a^ | 1974-1981 | 13.1[0.2,27.5] ^a^ |  | 1981-2014 | 1.1[0.5,1.7] ^a^ |  |  |  |
| Nasopharynx | Female | -2.7[-3.8,-1.5] ^a^ | 1974-2014 | -2.7[-3.8,-1.5] ^a^ |  |  |  |  |  |  |
|  | Male | -1.9[-5.2,1.4] | 1974-2012 | 0.3[-0.4,1.0] |  | 2012-2014 | -36.3[-67.8,26.0] |  |  |  |
| Larynx ^b^ | Male | 1.4[-4.6,7.7] | 1974-1999 | 3.9[1.7,6.1] ^a^ |  | 1999-2002 | -23.6[-65.5,69.5] |  | 2002-2014 | 3.3[-1.5,8.4] |
| Other thoracic organs ^b^ | Male | -0.4[-9.3,9.3] | 1974-1991 | -1.2[-6.3,4.1] |  | 1991-1994 | 35.8[-60.8,369.7] |  | 1994-2014 | -4.3[-7.4,-1.2] ^a^ |
| Bone | Female | -3.5[-9.0,2.4] | 1974-2003 | -0.5[-1.9,0.9] |  | 2003-2007 | -29.1[-57.7,18.7] |  | 2007-2014 | 1.5[-15.2,21.4] |
|  | Male | -2.7[-9.4,4.5] | 1974-2001 | -0.6[-2.1,0.9] |  | 2001-2004 | -30.7[-72.4,73.9] |  | 2004-2014 | 1.9[-7.8,12.5] |
| Kidney | Female | 1.7[0.4,2.9] ^a^ | 1974-2014 | 1.7[0.4,2.9] ^a^ |  |  |  |  |  |  |
|  | Male | 2.5[1.6,3.5] ^a^ | 1974-2014 | 2.5[1.6,3.5] ^a^ |  |  |  |  |  |  |
| Prostate | Male | 4.3[3.5,5.0] ^a^ | 1974-2014 | 4.3[3.5,5.0] ^a^ |  |  |  |  |  |  |
| Lymphoma | Female | -0.4[-1.3,0.4] | 1974-2014 | -0.4[-1.3,0.4] |  |  |  |  |  |  |
|  | Male | -0.1[-0.6,0.4] | 1974-2014 | -0.1[-0.6,0.4] |  |  |  |  |  |  |
| Ovary | Female | 1.3[0.0,2.6] ^a^ | 1974-1998 | 2.9[1.2,4.6] ^a^ |  | 1998-2014 | -1.0[-3.1,1.2] |  |  |  |

a. APC value is significantly different from Zero at alpha=0.05.

b. APC was not calculated in female population for age-standardized rate is zero in one year or more.
