# Supplementary material for: Cancer mortality trends in an industrial district of Shanghai, China, from 1974 to 2014, and projections to 2029
Source: Oncotarget. 2017 Sep 30;8(54):92470–82. doi: 10.18632/oncotarget.21419 (PMC5696197; doi:10.18632/oncotarget.21419)
Supplement: Supplementary file 4 [file oncotarget-08-92470-s004.docx]

**Supplementary Table 5: Projected number of cancer death during 2015-2019, 2020-2024, and 2025-2029 by sex**

| Cancer site | 2015-2019 | | |  | 2020-2024 | | |  | 2025-2029 | | |
| --- | --- | --- | --- | --- | --- | --- | --- | --- | --- | --- | --- |
|  | Number of death | Percent | Rank |  | Number of death | Percent | Rank |  | Number of death | Percent | Rank |
| Women |  |  |  |  |  |  |  |  |  |  |  |
| Lung | 1211.53 | 18.89% | 1 |  | 1038.33 | 18.45% | 1 |  | 626.24 | 17.85% | 1 |
| Breast | 646.7 | 10.08% | 3 |  | 612.67 | 10.89% | 3 |  | 431.24 | 12.29% | 2 |
| Colorectum | 785.44 | 12.25% | 2 |  | 689.28 | 12.25% | 2 |  | 417.97 | 11.92% | 3 |
| Pancreas | 563.98 | 8.79% | 4 |  | 492.05 | 8.74% | 4 |  | 277.91 | 7.92% | 4 |
| Stomach | 557.52 | 8.69% | 5 |  | 442.46 | 7.86% | 5 |  | 269.29 | 7.68% | 5 |
| Liver | 344.47 | 5.37% | 7 |  | 280.08 | 4.98% | 7 |  | 165.19 | 4.71% | 6 |
| Lymphoma | 245.16 | 3.82% | 8 |  | 240.15 | 4.27% | 8 |  | 159.96 | 4.56% | 7 |
| Gallbladder | 367.88 | 5.74% | 6 |  | 307.76 | 5.47% | 6 |  | 158.63 | 4.52% | 8 |
| Ovary | 224.05 | 3.49% | 9 |  | 186.56 | 3.32% | 9 |  | 111.96 | 3.19% | 9 |
| Esophagus | 144.08 | 2.25% | 11 |  | 143.35 | 2.55% | 11 |  | 98.09 | 2.80% | 10 |
| Leukemia | 173.41 | 2.70% | 10 |  | 146.38 | 2.60% | 10 |  | 88.62 | 2.53% | 11 |
| Cervix | 106.02 | 1.65% | 12 |  | 98.86 | 1.76% | 12 |  | 80.74 | 2.30% | 12 |
| Uterus | 100.1 | 1.56% | 13 |  | 92.5 | 1.64% | 13 |  | 63.49 | 1.81% | 13 |
| Other | 943.76 | 14.71% | / |  | 1038.33 | 15.22% | / |  | 558.19 | 15.91% | / |
| Men |  |  |  |  |  |  |  |  |  |  |  |
| Lung | 3139.52 | 31.75% | 1 |  | 2701.99 | 34.68% | 1 |  | 1663.31 | 30.25% | 1 |
| Colorectal | 1688.63 | 17.08% | 2 |  | 1747.08 | 28.72% | 2 |  | 1209.98 | 22.01% | 2 |
| Prostate | 656.11 | 6.64% | 5 |  | 770.13 | 5.62% | 3 |  | 515.82 | 9.38% | 3 |
| Stomach | 680.31 | 6.88% | 4 |  | 568.41 | 4.40% | 4 |  | 348.09 | 6.33% | 4 |
| Esophagus | 390.33 | 3.95% | 6 |  | 324.75 | 2.73% | 6 |  | 219.18 | 3.99% | 5 |
| Liver | 771.14 | 7.80% | 3 |  | 468.16 | 3.79% | 5 |  | 215.7 | 3.92% | 6 |
| Pancreas | 303.28 | 3.07% | 7 |  | 266.9 | 2.31% | 7 |  | 157.24 | 2.86% | 7 |
| Kidney | 250.98 | 2.54% | 9 |  | 248.31 | 2.20% | 8 |  | 151.11 | 2.75% | 8 |
| Lymphoma | 293.19 | 2.97% | 8 |  | 222.59 | 2.01% | 9 |  | 126 | 2.29% | 9 |
| Bladder | 215.05 | 2.17% | 10 |  | 188.35 | 1.74% | 10 |  | 104.39 | 1.90% | 10 |
| Brain,CNS | 180.64 | 1.83% | 12 |  | 141.41 | 1.35% | 12 |  | 81.02 | 1.47% | 11 |
| Leukemia | 191.19 | 1.93% | 11 |  | 143.8 | 1.35% | 11 |  | 80.75 | 1.47% | 12 |
| Others | 1127.83 | 11.41% | */* |  | 993.66 | 9.60% | / |  | 625.93 | 11.38% | / |
